# Presentation Attack Face Image Generation Based on a Deep Generative Adversarial Network

**DOI:** 10.3390/s20071810

**Published:** 2020-03-25

**Authors:** Dat Tien Nguyen, Tuyen Danh Pham, Ganbayar Batchuluun, Kyoung Jun Noh, Kang Ryoung Park

**Affiliations:** Division of Electronics and Electrical Engineering, Dongguk University, 30 Pildong-ro 1-gil, Jung-gu, Seoul 04620, Korea; nguyentiendat@dongguk.edu (D.T.N.); phamdanhtuyen@gmail.com (T.D.P.); kjn0908@naver.com (K.J.N.); parkgr@dongguk.edu (K.R.P.)

**Keywords:** generative adversarial network, presentation attack detection, artificial image generation, presentation attack face images

## Abstract

Although face-based biometric recognition systems have been widely used in many applications, this type of recognition method is still vulnerable to presentation attacks, which use fake samples to deceive the recognition system. To overcome this problem, presentation attack detection (PAD) methods for face recognition systems (face-PAD), which aim to classify real and presentation attack face images before performing a recognition task, have been developed. However, the performance of PAD systems is limited and biased due to the lack of presentation attack images for training PAD systems. In this paper, we propose a method for artificially generating presentation attack face images by learning the characteristics of real and presentation attack images using a few captured images. As a result, our proposed method helps save time in collecting presentation attack samples for training PAD systems and possibly enhance the performance of PAD systems. Our study is the first attempt to generate PA face images for PAD system based on CycleGAN network, a deep-learning-based framework for image generation. In addition, we propose a new measurement method to evaluate the quality of generated PA images based on a face-PAD system. Through experiments with two public datasets (CASIA and Replay-mobile), we show that the generated face images can capture the characteristics of presentation attack images, making them usable as captured presentation attack samples for PAD system training.

## 1. Introduction

### 1.1. Introduction to Face-Based Biometric System

Biometric models, such as fingerprints, faces, irises, and finger-vein models, have been widely used in high-performance systems for recognizing/identifying a person [1,2]. In addition, these recognition systems offer more convenience to users than conventional recognition methods, such as token- and knowledge-based methods [1]. However, with the development of digital technology, biometric recognition systems are facing an increasing threat from attackers using fake samples to successfully circumvent recognition systems. 

Face-based recognition systems are popular biometric recognition systems and have been used for a long time to recognize people [3,4,5]. This type of biometric is based on the fact that facial appearance can be used to easily distinguish people. To prevent attackers, presentation attack detection for face recognition (face-PAD) systems have been proposed; these typically use a collection of real and presentation attack (PA) face images to train a detection model [6,7,8,9,10,11,12,13,14,15]. The performance of such face-PAD systems has been shown to be strongly dependent on the training data, in which PA images are captured by simulating several limited types of attacking methods, such as the use of a photo, video display, or mask. The real images that are captured using real human faces represented in front of capturing devices (camera) and PA images inherit differences because of different subjects such as the distribution of illumination, reflection, and noises. However, with the development of technology, the presentation attack face images are become closer to real face images and can possibly deceive the face recognition system, making it fail. In addition, the attack methods are very diverse according to PA instrument (PAI) and attack procedure, such as the use of a three-dimensional (3D) masks instead of two-dimensional (2D) masks, the use of a high quality photo/video instead of a low quality photo/video, or the use of different types of photo or different equipment for displaying videos. As a result, it is difficult to collect a large amount of PA face sample images that simulate all possible types of attacking methods to train the systems. Consequently, the performance of face-PAD systems can be reduced and biased if faced with a new type of attacking method that has not been simulated in the training data during detector training. This is still an open issue and must be studied in more detail to enhance the security of face recognition systems.

### 1.2. Problem Definition

As explained in Section 1.1, face-PAD systems are necessary for enhancing the security level of face-based recognition systems. However, a high-performance face-PAD system requires a huge amount of training data (real and PA images) in which the PA images can simulate all possible attack methods and scenario. Unfortunately, this kind of data is hard to collect in a real system because the attack methods and presentation attack instruments are diverse and can change and become more sophisticated as the technology develops. To solve this problem, our study aims to artificially generate PA images that are close to the captured PA images by learning the presentation attack characteristics of available captured PA images and the fusion of the real and these PA images. Our study makes the following four novel contributions:

- This is the first attempt to generate PA face images based on a deep-learning framework. By learning the characteristics of real and PA images in a training dataset, our method can efficiently generate PA images, which are difficult to collect using conventional image collection methods due to the diversity of attack methods.
- By training our CycleGAN-based generation network using both captured real and PA images, we learn the characteristics of PA images in addition to the fusion of real and PA images. This approach can consequently help to fill the gap of missing PA samples caused by the diversity of attack methods.
- We propose a new measurement method to evaluate the quality of generated images for biometric recognition systems based on the use of a conventional face-PAD system and the dprime measurement.
- The code and pre-trained models for PA image generation are available as a reference to other researchers [16].

The remainder of this paper is organized as follows: In Section 2, we summarize works related to our study. In Section 3, the proposed method is described in detail along with several necessary preprocessing steps. Using the proposed method in Section 3, we performed various experiments using two public datasets (including CASIA [7] and Replay-mobile [9]) to evaluate the generated PA images, and the results are given in Section 4. Finally, we conclude our work and discuss future work in Section 5.

## 2. Related Works

As explained in Section 1, researchers have paid much attention to developing face-PAD systems to detect PA samples from face recognition systems to enhance their security [6,7,8,9,10,11,12,13,14,15]. Initially, they used several handcrafted image feature extraction methods to extract image features and detect PA samples by applying some classification method based on the extracted image features [6,8,10,11]. For example, color information [10], texture information extracted by local binary pattern (LBP) or dynamic local ternary pattern (DLTP) [6,11], and the defocus phenomenon [8] have been used for face-PADs. In [17], Benlamoudi et al. proposed a method that combined multi-level local binary pattern (MLLBP) and multi-level binarized statistical image features (MLBSIF) for face-PAD. In addition, they compared the detection accuracy of their proposed method with other six handcrafted-based methods using CASIA dataset. However, their detection performances were not sufficient because they were designed by expert knowledge of researchers alone, which can only reflect some limited aspects of the face-PAD problem. Recently, with the development of learning-based methods, especially deep-learning, the detection performance of face-PAD systems has significantly enhanced by using image features extracted by convolutional neural networks (CNNs) instead of the handcrafted image features. Nguyen et al. [15] used a stacked CNN-RNN network to learn deep representation of input face sequences. By combining the deep and handcrafted image features, they showed that the detection performance of sequence-based face-PAD system is greatly enhanced compared to the use of only handcrafted features. Recently, Liu et al. [18] proposed a deep tree learning method for face-PAD systems. By using the zero-shot learning technique, they made the face-PAD system more generalized for unknown attack methods. However, a common limitation of deep-learning-based methods is that they require a large amount of data to efficiently train the detection models, which is usually difficult to obtain because it requires much labor and cost. An additional problem with common face-PAD systems is that attack methods are diverse. As shown in previous studies [6,7,8,9,10,11,12,13,14,15], attackers can use various methods to attack a face recognition system depending on the PAI and approach used. Therefore, it is practically impossible to collect sufficient data to simulate all possible attack cases to train a detection model. One possible solution to this problem is an automatic PA face image synthesis and generation method.

With the development of deep-learning frameworks, image generation has attracted many researchers in the computer vision research community [19,20,21,22,23,24,25,26,27,28,29,30,31,32]. A major method for generating images is the generative adversarial network (GAN), which was proposed by Goodfellow et al. [19]. This type of image generation method has successfully been applied to many computer vision tasks, such as image editing [20,21], super-resolution interpolation [22,23,24], image de-blurring [25], data generation [26,27,28], image-to-image translation [29,30,31], and attention prediction [32]. The key to the success of the GAN is that it trains two deep CNNs (the discriminator and generator) in an adversarial manner. Specifically, the discriminator network is responsible for discriminating between two classes of image, ‘real’ or ‘fake’, while the generator is responsible for generating ‘fake’ images that are as close as possible to ‘real’ images. These two networks are trained to perform their functionalities using a large amount of training data. As a result, the generator can generate fake images that are very similar to real images. 

For biometric image generation, there have been studies that generate images for palm-prints [26], irises [27], and fingerprints [28,33]. In [26], the authors used a GAN method to generate realistic palm-print images from a training dataset. In principal, a deep-learning-based system requires a large amount of training data to successfully train a network. However, collecting such data usually requires much effort and expense and sometimes is impossible due to the diversity of input images. To reduce the effects of this problem, several simple techniques, such as cropping-and-scaling, adding noise, and mirroring have been adopted to make the training dataset slightly more generalized. However, these simple methods are not strong enough for full data generalization. As a result [26], the GAN-based method is sufficient for generating realistic palm-print images and consequently helped to reduce the error of the palm-print recognition systems. For other biometric systems, such as the fingerprint and iris, a GAN-based network has also been used to generate images that are close to captured ones [27,28]. In [33], Bontrager et al. showed that fingerprint images generated by a GAN-based network can be used to fool fingerprint recognition systems. This means that the generated images were very similar to actual captured images and also demonstrates that fingerprint recognition systems are vulnerable.

Although GAN-based methods have been widely used for image generation problems, there have been no studies conducted to generate PA images for a face-PAD system. Inspired by the problem of face-PAD systems, we propose a PA image synthesis/generation method for the face-PAD problem based on a GAN. Our study serves two purposes. First, we aim to generate realistic PA images to reduce the effort of PA image acquisition in designing face-PAD systems. Secondly, by training our generation system using not only captured PA face images but also real face images, we tend to generate more trustable PA images, which can fill the gap of missing samples caused by the diversity of attacking methods. Table 1 summarizes the various previous studies that are related to ours.

## 3. Proposed Method

In this section, we provide a detailed description of our proposed method for generating PA face images using the deep-learning method based on the CycleGAN network architecture.

### 3.1. Overview of the Proposed Method

**Figure 1:**
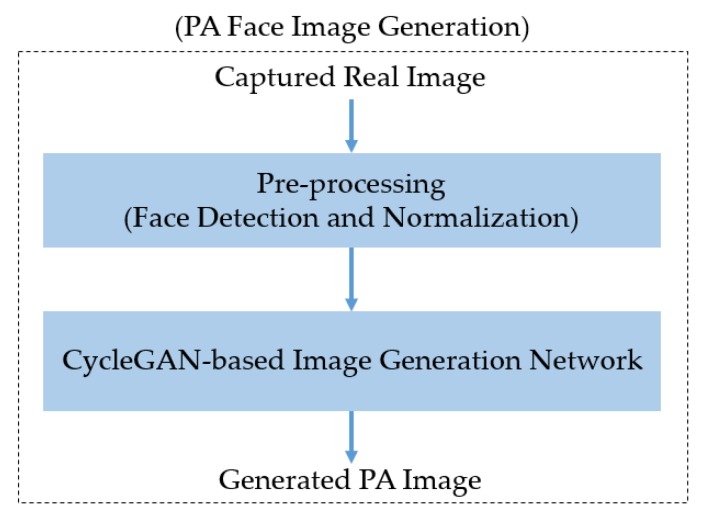
Overview of our proposed method for generating PA face images.

Figure 1 presents an overview of our proposed method for generating PA images. 

As explained in Section 2, our method aims to generate PA images using a Cycle-GAN network architecture. Therefore, the input of the network is a face image captured in the wild. To learn the characteristics of real and PA images efficiently for the generation problem, the input captured face images are first preprocessed by a face detection and normalization block to detect the face as well as align the face images. This step is explained in more detail in Section 3.2. Using the result of this step, we generate PA images using an image generation network, which is described in Section 3.3.

### 3.2. Face Detection and Normalization

As shown in Figure 1, our proposed method receives a captured face image as input and generates a PA image at the output. As indicated in previous studies [6,7,8,9,10,11,12,13,14,15], the discrimination information between real and PA images mostly appears inside the face region rather than in the background regions. In addition, the purpose of our proposed method is to generate realistic PA images to reduce the effort required in presentation attack image acquisition. Therefore, the input captured face image must be preprocessed to remove the background before using it to generate a PA image.

**Figure 2:**
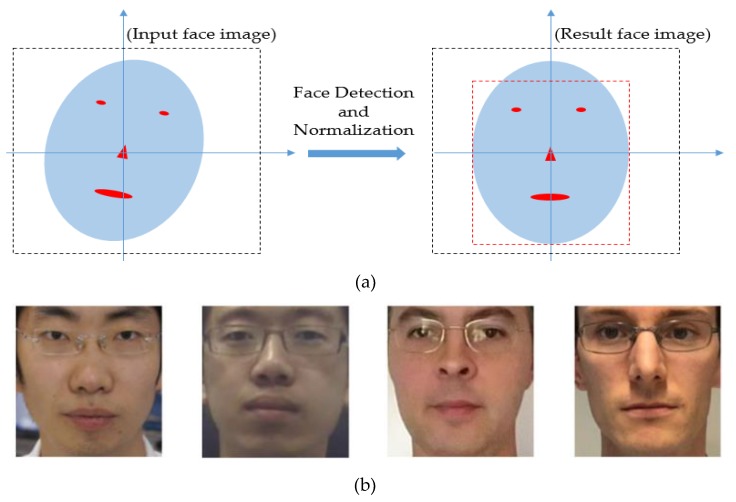
Methodology and an example result of face detection and misalignment compensation steps: (**a**) overview of method for face detection and in-plane rotation compensation with input captured face image (left) and result image (right); (**b**) example results.

Generally, an input captured face image contains not only faces but also background regions. Therefore, we perform two preprocessing steps on the input face image: face detection and in-plane rotation compensation [15]. As the first step, we use a face detection method based on the ensemble of regression tree (ERT) proposed by Kazemi et al. [34]. This is an efficient and well-known method for accurate face and face landmark detection. Using this method, we can efficiently locate the face and additional 68 landmark points on the detected face, which can be used to define face shape [15]. Because we are generating PA images by learning the discrimination information between two types of face images, the input face image should be aligned to reduce complexity and misalignment and to let the generator focus on learning the characteristics of the two types of images. Using the detected face and its landmark points, we further perform an in-plane rotation compensation step to compensate the misalignment of the input face image [15]. Figure 2 illustrates the methodology of these steps. In Figure 2a, we illustrate the abstract methodology of the face detection and in-plane rotation compensation steps. A detailed explanation of the mathematical and implementation techniques is provided by Nguyen et al. [15]. In Figure 2b, we show some example results (extracted face images) of the implementation of these steps. As shown in this figure, the final detected face images are aligned to a frontal face without in-plane rotation. This normalization step helps to reduce the effects of non-ideal input images and makes the training procedure more focus on learning the characteristics of images.

### 3.3. GAN-Based Image Generation Network

**Figure 3:**
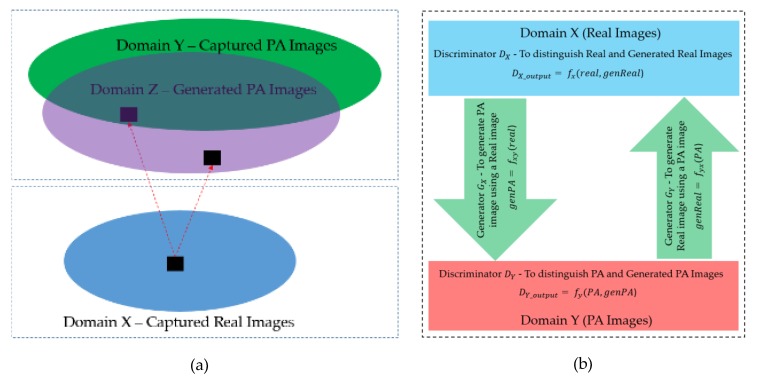
Overview of our deep-learning-based image generation network: (**a**) Demonstration of the distribution of real and PA images; (**b**) Overview of our network structure for PA image generation. In this figure, the *real* and *PA* indicate the captured real or presentation attack images; *genReal* and *genPA* indicate the generated real and generated presentation attack images using generator networks and the input *real* and *PA* images; $f_{x},f_{y},f_{xy},\;and\;f_{yx}$ indicates the relation function models by discriminator ($D_{X}$, $D_{Y}$) and generators ($G_{X}$, $G_{Y}$), respectively.

As explained in the above sections, our study aims to generate PA images using captured real images as the input of the generation network. Figure 3 illustrates the generation methodology of our study. In Figure 3a, we show a general concept of the distribution of the real and PA images in which the real images are captured by real human faces presenting in front of capturing devices, while the PA images can be obtained by either capturing presentation attack models (photo, video, mask) or generating by an image generation model. In Figure 3b, we show the general concept of our PA image generation framework. As shown in this figure, we can use captured real and PA images to train a generation model to learn the characteristics of these two types of face images and a transformation function from real to PA classes. As a result, we obtain a model to transform a captured real image into a PA image. To construct a generation model, we built a GAN network based on a popular image generation network, namely CycleGAN (as shown in Figure 3b), using two discriminators ($D_{X}$ and $\;D_{Y}$), which are responsible for distinguishing the real and generated real images ($D_{X})$ and distinguishing PA and generated PA images ($D_{Y})$, and two generators ($G_{X}$ and $\;G_{Y}$), which are responsible for generating PA images using real images ($G_{X})$ and generating real images using PA images ($G_{Y})$. The two discriminator networks share the same discriminator architecture, as described in Section 3.3.1, and the two generator networks share the same generator architecture, as described in Section 3.3.2.

To best of our knowledge, our study is the first attempt to use CycleGAN-based network to generated PA face images for face-PAD system. The reason for the use of a CycleGAN-based network to generate PA images in our study is that we tend not only to generate trustable PA images that are close to captured real images as much as possible but also to stimulate new type of presentation attacks that does not exist in training data. For this reason, we should learn the characteristics of both captured real and PA images, and the transformation between the two domains. Because of this reason, we think the CycleGAN-based network is most suitable for our goal.

#### 3.3.1. Discriminator Architecture

**Figure 4:**
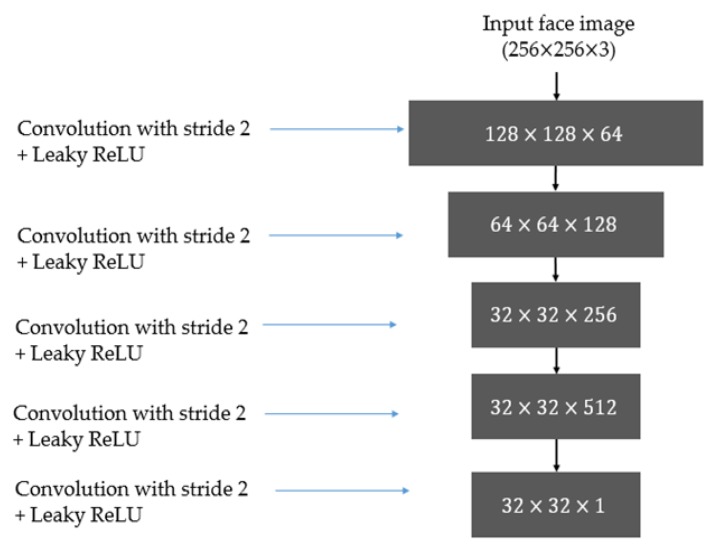
Illustration of the discriminator network used in our study.

The discriminator in the GAN-based network has the responsibility of distinguishing images in one class from images in another. Therefore, a discriminator is essentially a classification network. Inspired by the success of recent deep-learning-based image classification works [35,36,37,38,39,40,41,42], we use a conventional CNN to construct discriminators in our study. In previous studies on the image classification problem [35,36,37,38,39,40,41,42], a deep-learning-based network has been constructed using two separated parts, including convolution layers and fully-connected (dense) layers. Among these parts, the convolution layers are used to extract image features using the convolution operation. Based on the extracted image features, the fully connected layers are used to learn a classifier to classify input features into several predesigned groups. The use of these two parts leads to a high performance in classification systems. However, it has a weakness that it requires a huge number of parameters, which make the classifier complex and difficult to train. To reduce the effects of this problem, we design the discriminators in our GAN-based network by simply using convolution layers to extract image features. As a result, the image classification step is directly executed by comparing the extracted image features with the ground-truth image features of the desired class. In Figure 4, we illustrate the structure of the discriminator used in our study. As shown in this figure, the discriminator contains five convolution layers with a stride value of 2, followed by a leaky rectified linear unit (Leaky ReLU) as the activation function. This network accepts an input color image of 256-by-256 pixels to produce a feature map of 32-by-32 pixels as the output. Table 2 gives a detailed description of the layers and their parameters in the discriminator network. Although pooling layers, such as max or average pooling, are frequently used after convolution layers for feature selection to make the CNN network less invariant to image translation, this is not suitable in our case, which uses only the convolution operation in the discriminator. The reason for this is that pooling layers select a dominant feature for each image patch. As a result, the extracted feature maps are misaligned and give poor classification accuracy. To solve this problem, the discriminator in our study only uses convolution layers with a stride of 2 without a pooling layer. This implementation helps to extract image features in patches (blocks of image) and removes the effects of misalignment on the extracted feature maps. 

In our study, we only use the convolution layers to extract image features (feature maps) and matching the output of the network with the corresponding label feature maps where feature map of ones represents the ground-truth label of real images, and feature map of zeros represents the ground-truth label of PA images. Therefore, Figure 4 does not contain some layers such as fully connected layer (FC), softmax, or classification. Although we can add these layers (FC, softmax, etc.) to the end of this figure to construct a discriminator as what has done with a normal convolutional neural network, the use of only convolutional layers helps to reduce the number of network parameter and make it not depend on the shape of input images. Consequently, it helps to reduce the overfitting problem that normally occurs in training the deep-learning-based networks.

As shown in Table 2, our discriminator network uses an even kernel in convolution layers. Although odd kernels have been normally used in CNN networks, the even kernel has been used in previous studies for GAN models such as conditional GAN [20], de-blurred GAN [25], pix2pix [31], or CycleGAN-based network [29]. Therefore, we selected to use even kernels in our study.

#### 3.3.2. Generator Architecture

**Figure 5:**
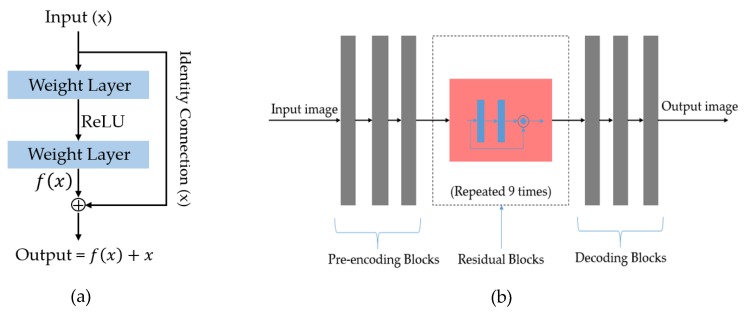
Illustration of the generator network used in our study: (**a**) residual block architecture that is used to increase depth of network; (**b**) draw sketch structure of generator network.

The generator, which is responsible for image generation, is the heart of a GAN-based network. In our study, we use a deep CNN to construct the generator. In detail, the generator is constructed as an auto encoder-decoder network, as shown in Figure 5. At the input, the generator accepts an input image and then performs initial pre-encoding steps, which are composed of three convolution layers to encode the input image. As indicated in previous studies [37,41,42,43], the performance of deep-learning-based systems can be much enhanced by making them deeper. Using this characteristic, we continue processing the feature maps by applying a sequence of nine residual blocks to further manipulate the image data. 

The residual connection was first introduced in the work by He et al. [42]. In their work, they showed that it is difficult to train a very deep neural network that is made by linearly stacking weight layers. The problem is caused by the vanishing gradient problem that normally occurs when the depth of the network increases. Consequently, this problem causes the network’s performance saturated and degrading rapidly. To solve this problem, He et al. proposed a new network structure, namely residual block, as shown in Figure 5a, where the residual block uses an identity shortcut connection to skip one or more layers during training the network. As a result, training of some layers becomes easier if they simply are identity mappings. 

Based on this architecture, we use the residual block in our study to increase the depth of the generator network that is efficient for enhancing the performance of a neural network. The reason is that we not only want to make the network deeper but also make the network easy to train using a residual architecture, which was successfully designed to reduce the effects of the exploring/vanishing gradient problem [42]. To obtain the output image, we use several deconvolution layers on the output of the residual blocks, as shown in Figure 5b. In Table 3, we give a detailed description of the generator network used in our study.

In Table 2 and Table 3, the “Normalization” implies the instance-normalization that normalizes the feature maps to a normal distribution with zero-mean and unit variance [25]. The normalization technique is normally used in the neural network to normalize feature maps and make them in the same range and comparable. For the generator network, we tend to generate a real image that normally has pixel values in the range of [0,255] (color image) or [−1, 1] (normalized image) at the output of the network. Therefore, we used the Tanh normalization function at the last layer of the generator to scale the output of the network in the range from −1 to +1.

#### 3.3.3. Calculation of Loss

As discussed in the above sections, our proposed method is based on the CycleGAN network architecture for training an image generation model. To train our network, we must calculate the values of the loss function during the training procedure. For this purpose, let X and Y be two image domains corresponding to the “real” and “PA” classes, respectively. As shown in Figure 3b, we use two discriminator networks ($D_{X\;}$ and $\;D_{Y}$) and two generator networks ($G_{X}$ and $\;G_{Y}$) to construct the GAN network in our study. In each domain, we have one generator and one discriminator, as shown in Figure 3b. In detail, the discriminator $D_{X}$ is the discriminator in the X domain, which is responsible for discriminating samples in the X domain from those generated by $G_{Y}$ using samples in the Y domain; the generator $G_{X}$ is the generator in the X domain, which is responsible for generating samples in the Y domain using input samples in the X domain. Similarly, we have the discriminator $D_{Y}$ and generator $G_{Y}$ in the Y domain. $G_{X}$ is used to generate fake samples of the Y domain using samples in the X domain, and $D_{Y}$ is used to discriminate the ground-truth samples of the Y domain from those generated by $\;G_{X}$. Therefore, we define the adversarial loss function $L_{GAN}\left(G_{X},D_{Y},X,Y\right)$ as follows:

(1) $$L_{GAN}\left(G_{X},D_{Y},X,Y\right)=E_{y\sim p\left(y\right)}\left[log\left(D_{Y}(y\right)\right]+{\;E}_{x\sim p\left(x\right)}\left[log\left(1-{\;D}_{Y}\left(G_{X}\left(x\right)\right)\right)\right]$$

The first term $E_{y\sim p\left(y\right)}\left[f\left(D_{Y}(y\right)\right]$ is the mean of the loss of the discriminator $D_{Y}$ using ground-truth samples in the Y domain (PA images), and the second term $E_{x\sim p\left(x\right)}\left[log\left(1-D_{Y}\left(G_{X}\left(x\right)\right)\right)\right]$ is the mean of the loss of $D_{Y}$ using the PA images generated by $G_{X}$ because $D_{Y}$ is responsible for discriminating its ground-truth sample (PA image) from the samples generated by $G_{X}$. Similarly, we have the adversarial loss $L_{GAN}\left(G_{Y},D_{X},Y,X\right)$ for $G_{Y}$ and $D_{X}$ as follows:

(2) $$L_{GAN}\left(G_{Y},D_{X},Y,X\right)=E_{x\sim p\left(x\right)}\left[log\left(D_{X}(x\right)\right]+{\;E}_{y\sim p\left(y\right)}\left[log\left(1-{\;D}_{X}\left(G_{Y}\left(y\right)\right)\right)\right]$$

Equations (1) and (2) describe the loss function using the conventional cross-entropy loss function. However, as indicated by previous research [44], the use of the standard cross-entropy loss function in a deep convolutional GAN (DCGAN) can cause the vanishing gradient problem, and this problem makes the network difficult to train. To overcome this problem and make network training easier, we use the least-squared error instead of conventional cross-entropy for loss calculation in our experiments. As a result, the adversarial loss is as described by Equations (3) and (4) as follows:

(3) $$L_{GAN}\left(G_{X},D_{Y},X,Y\right)=E_{y\sim p\left(y\right)}\left[{\left(D_{Y}\left(y\right)-1\right)}^{2}\right]+{\;E}_{x\sim p\left(x\right)}\left[{\left(D_{Y}\left(G_{X}\left(x\right)\right)\right)}^{2}\right]$$

(4) $$L_{GAN}\left(G_{Y},D_{X},Y,X\right)=E_{x\sim p\left(x\right)}\left[{\left(D_{X}\left(x\right)-1\right)}^{2}\right]+{\;E}_{y\sim p\left(y\right)}\left[{\left(D_{X}\left(G_{Y}\left(y\right)\right)\right)}^{2}\right]$$

In addition to the adversarial losses, we also use cycle-consistent-loss (cycle-loss) in the reconstructed path to ensure the quality of the reconstruction of the input images using the two generator networks $G_{X}$ and $\;G_{Y}$. Cycle-loss is defined by Equation (5):

(5) $$L_{cycle}\left(G_{x},G_{y}\right)={\;E}_{x\sim p\left(x\right)}\left[||G_{y}\left(G_{x}\left(x\right)\right)-x||\right]+{\;E}_{y\sim p\left(y\right)}\left[||G_{x}\left(G_{y}\left(y\right)\right)-y||\right]$$

As a result, the final loss function used in our study is given by Equation (6), which takes a weighted sum of the adversarial loss and cycleloss. In this equation, λ is used to indicate the weight (importance) of cycleloss over adversarial loss. In our experiments, we used the lamda value of 10 as suggested by Zhu et al. [29].

(6) $$L_{GAN}\left(G_{X},G_{Y},D_{X},D_{Y}\right)=L_{GAN}\left(G_{X},D_{Y},X,Y\right)+L_{GAN}\left(G_{Y},D_{X},Y,X\right)+{\;\lambda L}_{cycle}\left(G_{x},G_{y}\right)$$

## 4. Experimental Results

### 4.1. Experimental Setups

As explained in our above sections, our study purpose is to generate PA images those are close to the captured PA images, not detecting PA images. Similarity measurements are usually used to evaluate the performance of such systems. As indicated by their meaning, a high performance image generation system has the ability to generate images which are as similar as the ground-truth images. To measure the performance of the image generation model, we followed a well-known quality measurement, namely Frechet Inception Distance (FID) [27,28,45,46,47,48]. In addition, we proposed a new quality measurement, namely presentation attack detection distance (padD) as shown in Equation (8). This newly proposed measurement has nice graphical visualization characteristics and it is customized for face-PAD problem. Along with padD measurement, we additionally measured the distribution and the error of face-PAD method with newly generated PA images using attack presentation classification error rate (APCER) measurement, which is followed the ISO/IEC JTC1 SC37-ISO/IEC WD 30107-3 standard for presentation attack detection [49]. The APCER which is along with bona-fide classification error rate (BPCER) and average classification error rate (ACER) are the three popular performance measurements for a presentation attack detection system defined in ISO/IEC JTC1 SC37 standard [49]. By measuring the APCER value, we can evaluate the probability of a generated PA image successfully circumvent a face-PAD system.

For the first measurement method, the FID score is used to measure the quality of the generated images based on the features extracted by a pretrained inception model [43]. This method was proved to work better than the traditional inception score (IS) method [45]. In detail, this method compares the similarity of the two distributions of the extracted features of captured PA images and those of the generated PAD images. For this purpose, a pretrained inception model, which was successfully trained using the ImageNet dataset, is used to extract a 2048-dimensional feature vector for each input image. Suppose that we have N captured PA images and M generated PA images. Using this method, we extract N and M feature vectors for captured PA and generated PA images, respectively. Because the N captured PA images are from the same class (the ground-truth PA images), they form a distribution in a 2048-dimensional space. A similar situation occurs with the M generated images. As a result, we have two distributions for the two classes of images. Suppose these distributions are normal distributions with mean μ and covariance matrix Σ. Then, the FID is given by Equation (7):

(7) $$\mathrm{FID}=||\;\mu_{r}-\mu_{g}||{}^{2}+Tr\left(\Sigma_{r}+\Sigma_{g}-2{\left(\Sigma_{r}\Sigma_{g}\right)}^{\frac{1}{2}}\right)$$

In this equation, the subscripts r and g represent real and generated images, respectively. As shown in Equation (7), the FID measures the dissimilarity between the two distributions in 2048-dimensional space. As a result, a small value of FID indicates a high level of similarity between the two distributions. The FID measurement is based on the texture features extracted from images using a general deep-learning-based feature extraction model (a pre-trained inception model). Therefore, it can be used to assess the quality of the generated image in general. For our specific case of generating PA images for a face-PAD system, we suggest the use of an additional measurement to assess the quality of the generated PA images based on the use of an actual pretrained face-PAD system instead of the inception model, i.e., padD. The concept of the padD measurement is similar to that of the FID, but it uses a different feature extraction method. Because a face-PAD system is designed to discriminate real and PA images, well-generated PA images should have similar characteristics to captured PA images using a face-PAD system. Usually, a face-PAD system receives an image (or sequence of images) to produce a detection score, which stands for probability of the input image belonging to the real or PA class. If the output score is greater than a predefined threshold, the input image is regarded as a real image. Otherwise, the input image is regarded as a PA image. Using the N captured and M generated PA images, we can obtain N and M detection scores for the captured and generated PA image classes, respectively. Finally, we measure the dprime value of the two distributions, as given by in Equation (8), and use this value as the quality measurement of the generated PA images:

(8) $$\mathrm{padD}=\frac{\left|mean_{r}-mean_{g}\right|}{\sqrt{\frac{\left(\sigma_{r}^{2}+\sigma_{g}^{2}\right)}{2}}}$$

As shown in Equation (8), padD measures the distance between the two distributions in one-dimensional space using the dprime method based on two means and standard deviations. As a result, padD is large if the two distributions are very different and becomes smaller as the two distributions become more similar. We can see that padD is a custom FID measurement that is specialized for face-PAD systems. By using both the FID and padD values, we can assess the quality of generated images in more detail. In our experiments, we use two face-PAD systems for measuring the padD value: a deep-learning-based and a handcrafted-based face-PAD system. The deep-learning-based face-PAD system uses a combination of a CNN, recurrent neural network (RNN), and a multi-level local binary pattern (MLBP) for feature extraction and support vector machine (SVM) for classification. The handcrafted-based face-PAD system uses only the MLBP for feature extraction and SVM for classification [50].

To evaluate the performance of our proposed method, we perform experiments using two public datasets: CASIA [7] and Replay-mobile [9]. A detailed description of each dataset is given in Table 4 and Table 5, respectively. Originally, these datasets were widely used for training face-PAD systems [7,9,13,15]. The difference between the two datasets is that the CASIA dataset was created for the face-PAD problem in general using a normal camera, while the Replay-mobile dataset is specialized for the mobile environment. As shown in Table 4, the CASIA dataset contains captured real and PA images of 50 persons stored in video format. In total, the CASIA dataset contains 600 video clips (12 video clips (3 real attacks and 9 PAs) per person). By using the face detection method in Section 3.2, we extracted a total of 110,811 face images for the CASIA dataset. The advantage of the CASIA dataset is that it simulates rich attacking methods, including three levels of image resolution (low, normal, and high) and three methods for making PA samples (cut-photo, wrap photo, and video display). As shown in Table 4, the CASIA dataset is pre-divided into training and testing sub-datasets by the dataset’s owner for training and testing purposes.

The Replay-mobile dataset contains real and PA images of 40 persons from a mobile camera [9]. This dataset is also pre-divided into three sub-datasets for training, testing, and validation. However, we only use the training and testing datasets in our experiments because we do not need to validate the generation model. For both datasets, we use the training dataset to train the generation model and the testing dataset to measure the quality of the generated images.

### 4.2. Results

As explained in Section 1, the goal of our study is to construct a method for efficiently generating PA images to save efforts in collecting PA images in training a face-PAD system. For this purpose, in this section, we perform various experiments using two public datasets, i.e. CASIA and Replay-mobile, to evaluate the performance of our proposed method in comparison with previous studies. In summary, we first train our proposed image generation models mentioned in Section 3 using these two datasets and the results are presented in this section. Using these trained models, we further evaluate the quality of generated images using two quality measurements, i.e. FID and padD. Finally, we measure the processing time of the image generation model in two hardware systems, including a desktop computer and an embedded system based on an NVIDIA Jetson TX2 board to demonstrate the ability of our proposed method in a real application.

#### 4.2.1. Quality Assessment of Generated Images Using FID Measurement

**Figure 6:**
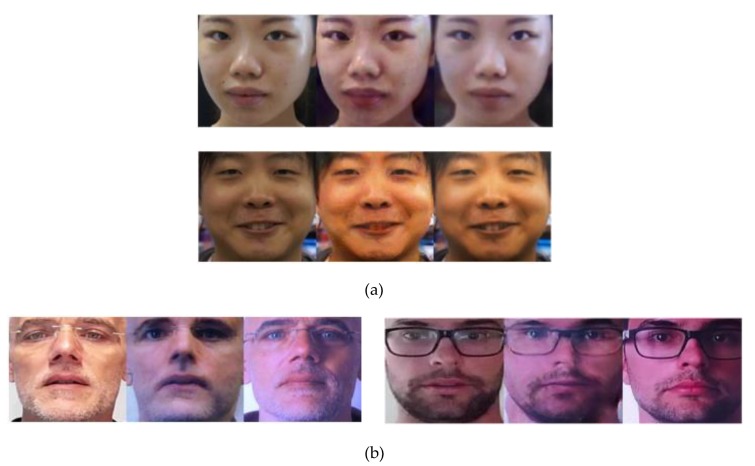
Examples of generated images using the trained generation model: (**a**) Images in the CASIA dataset. (**b**) Images in the Replay-mobile dataset. In each trio of images, the left, middle, and right images represent the real image, generated PA image, and reference PA image, respectively.

We show some example result images in Figure 6. In this figure, the left images are captured real images (the input of generation model), the middle images are the corresponding generated PA images, and the right images are reference captured PA images of the same person. As shown in this figure, the generation model can efficiently generate PA images using the captured real images by adding additional effects on the face, such as noise, blurring, color change, and textures. Although these effects can be added to images using conventional methods (adding noise, performing blurring, etc.), they are not manually added but learnt from the captured images in-the-wild. Therefore, we believe that the generated images are more appropriate than the ones using conventional methods.

In the next experiment, we evaluated the quality of generated images using the FID measurement mentioned in Section 4.1. For this purpose, we applied the trained generation model to the CASIA and Replay-mobile testing datasets. Because there is no previous research on this problem, we do not know whether the measured FID in our experiment is good or not. To solve this problem, we additionally measured the FID values between the captured PA images. Because the captured PA images are images captured in-the-wild by simulating attacking methods, they are correct PA images, and measuring the FID between two sets of captured PA images gives us a criterion for evaluating the performance of the generation model. We refer to the FID between the two subsets of captured PA images as the intra-class FID and to the FID between the captured PA images and generated PA images as the inter-class FID in this study.

To measure the intra-class FIDs, we used two different sets of captured PA images: one from the captured PA images in the training dataset and the other from the captured PA images in the testing dataset. This selection ensures two things. First, the images of the two sets are different but cover similar characteristics of PA images as they are from training and testing datasets. Secondly, the size of each set is as large as possible. Even if we divided the captured PA images of either the training or testing dataset into two subsets and measure the FID between these two sets, the number of images in each set would be reduced. As a result, the population of PA images would smaller than it is using our method. For the inter-class FID, we first generated PA images using the captured real images from the testing dataset. With the generated images, we performed the FID measurement using the captured PA images in the testing dataset. The detailed experimental results from the CASIA and Replay-mobile datasets are given in Table 6. As shown in this table, the intra-class FID of the CASIA dataset is approximately 24.614, while the intra-class FID of the Replay-mobile dataset is approximately 37.943. These two FID values are relatively different because the PA images from the two datasets are different. While the CASIA dataset was collected using a commercial camera in good illumination, the Replay-mobile dataset was collected using a mobile camera with uncontrolled light conditions. As a result, the variation of PA face images in the Replay-mobile dataset is large, which resulted in the high intra-FID value. Using the generated images, we obtained an inter-class FID for the CASIA dataset of approximately 28.300, and that of the Replay-mobile dataset was approximately 42.066. Because the intra-class FID was obtained from the ground-truth captured PA images, we can estimate that the intra-class FID should be lower than the inter-class FID because the inter-class FID was obtained using generated PA images. From Table 6, it can be seen that the differences between the intra-class FID and inter-class FID for the CASIA and Replay-mobile datasets are not too high (24.614 vs. 28.3 for the CASIA dataset and 37.943 vs. 42.066 for the Replay-mobile dataset). 

In addition, we performed experiments using the conventional cross-entropy loss function for a CycleGAN-based image generation model and compared its performance with the least-squared loss function. For this purpose, we measured the FID value between the captured PAD and generated PAD images obtained by a cross-entropy-based CycleGAN model. As explained in Equation (7) of Section 4.1, smaller FID means the higher performance of image generation model. The detail experimental results are given in Table 6. As shown in Table 6, we obtained an FID of 30.968 using a cross-entropy-based CycleGAN model which is larger than the 28.300 using the least-squared-based CycleGAN model with CASIA dataset. Similarly, we obtained an FID of 51.207 using the cross-entropy-based CycleGAN model which is larger than the 42.066 using the least-squared-based CycleGAN model with Replay-mobile dataset. These results confirmed that the least-squared loss function is better than the conventional cross-entropy loss function in our experiments.

As explained in Section 2, there have been previous studies that generated images between two different domains. Popular methods are DCGAN [27,28], the pix2pix [31], CycleGAN [29], and DualGAN [51] networks. To the best of our knowledge, the pix2pix [31] GAN network requires pairwise images (one for input image, and the other one for ground-truth label image) for learning the relation between the two domains. Therefore, it is not suitable for applying to our study because we are transforming the images between two domains (real vs. PA) without information of pairwise images. The DualGAN [51] is another option (beside CycleGAN) that could be suite for our problem. However, the methodology and structure of DualGAN and CycleGAN is very similar. Therefore, we compared the performance of image generation using DCGAN-based network with our proposed CycleGAN-based method. The experimental results are given in Table 7.

In Table 7, we give a comparison between the FIDs measured in our study and those from previous studies which use DCGAN for image generation problem. Minaee et al. [27] used a GAN to generate iris images. In their study, they showed that the FIDs between the ground-truth and generated images were approximately 41.08 on the IIT-Delhi dataset and 42.1 on the CASIA-1000 dataset. Similarly, the authors of [28] showed that the FID between the ground-truth and generated fingerprint images was approximately 70.5 using a GAN--based method. We can see that the FIDs obtained by our study are much smaller than those obtained by previous studies. Although it is unbalanced to compare the FIDs among different biometrics models because of the difference of image characteristics, we can roughly conclude that our results are comparable or better than those of previous studies.

For ensure a fair comparison, we additionally performed experiments for PA image generation using a DCGAN model. For this purpose, we trained a DCGAN model [27,28] using the CASIA and Replay-mobile datasets and measured the FID between the captured PA and DCGAN-based generated PA images as shown in Table 7, where we obtained an FID of 65.049 in the case of using the captured and generated PA images using DCGAN and the Replay-mobile dataset. This value is much bigger than that of 42.066 using the proposed method. Similarly, we obtained an FID of 82.400 for the case of DCGAN trained on the CASIA dataset. This FID measurement is also much bigger than 28.300 using our proposed method.

Based on these experimental results, we conclude that our proposed method can generate realistic PA images. In addition, the Cycle-GAN-based method is more sufficient than DCGAN-based method, and the Cycle-GAN-based network is a sufficient choice to solve our problem.

#### 4.2.2. Quality Assessment of Generated Images Using padD Measurement on CASIA Dataset

FID measurements have been widely used to evaluate the quality of generated images in general using deep features extracted by a pretrained inception model, which was successfully trained for the general image classification problem. Therefore, FID measurements seem to be too general for our problem. As explained in Section 4.1, our study proposes a new criterion for assessing the quality of generated PA face images called padD. The purpose of this new measurement is to evaluate the quality of generated images for the specific problem of PA image generation. For this purpose, we used an up-to-date face-PAD system [15] to generate decision scores of captured and generated PA images and measure the distance between the two score distributions of these two classes. As a result, if the two distributions are close each other, the generated images have similar characteristics to the captured images. Otherwise, the generated images are different from the captured PA images. One important characteristic of the padD measurement is that it allows a graphical visualization of the distributions of the ground-truth and generated images, which that is not available with the FID. This is because we are working with a one-dimensional feature space instead of a 2048-dimensional feature space. Therefore, the padD measurement gives us a more intuitive measurement than the FID.

**Figure 7:**
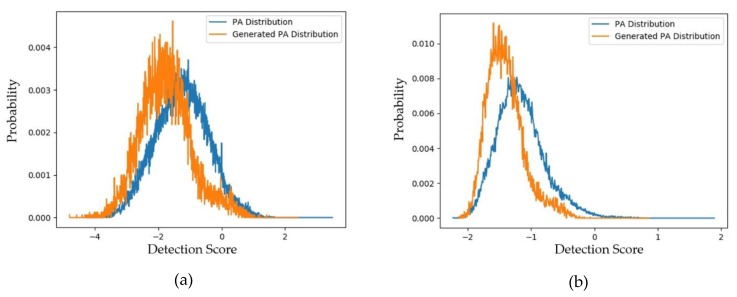
Distribution of detection scores for captured PA images versus the generated PA images with the CASIA dataset (**a**) using the handcrafted-based face-PAD system [50]; (**b**) using the deep-learning-based face-PAD system [15].

As the first experiment in this section, we measured the distributions and padD values for the case of using captured and generated PA images using both face-PAD systems (deep-learning-based and handcrafted-based method). The experimental results are given in Figure 7a,b for the handcrafted-based and the deep-learning-based face-PAD systems, respectively. The specific padD values are listed in Table 8. As shown in Figure 7, the distribution of captured PA images is relatively similar to that of the generated PA images. Numerically, Table 8 shows that the distance (padD) between the two distributions in Figure 7a is approximately 0.610 and that in Figure 7b is approximately 0.711.

**Figure 8:**
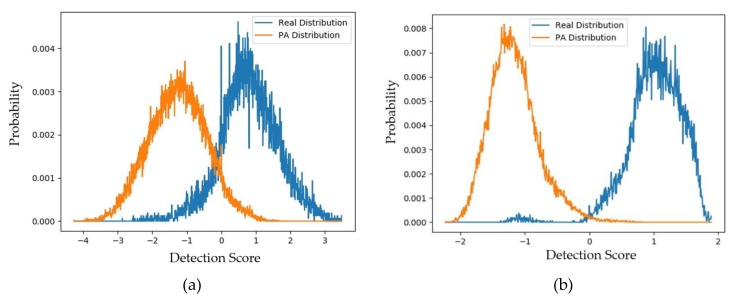
Distribution of detection scores for the original captured data (captured real and captured PA images): (**a**) results obtained using handcrafted-based face-PAD system [50]; (**b**) results obtained using deep-learning-based face-PAD system [15].

To evaluate these above padD measurements, we additionally measured the distributions and padD values for the original (captured real and PA images) CASIA dataset. Figure 8a,b show the distributions of the captured real and captured PA images using the CASIA testing dataset for the handcrafted-based and the deep-learning-based face-PAD systems, respectively. From this figure, it can be observed that the distributions of captured real and PA images were relatively separated. As a classification problem, the errors of this face-PAD system were approximately 0.910% and 9.488% for the deep-learning-based and handcrafted-based method, respectively. As indicated in [15], the error produced by the deep-learning-based method is the smallest compared to other previously proposed face-PAD systems using the CASIA dataset. 

**Figure 9:**
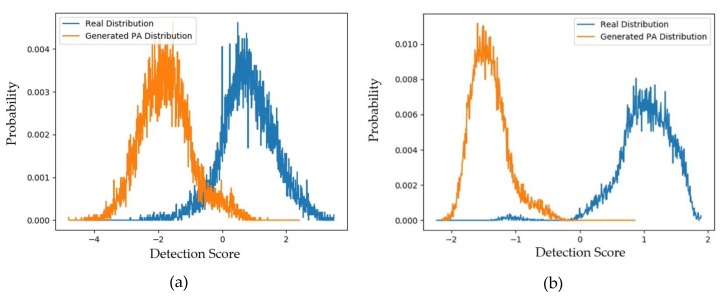
Distribution of detection score of captured real and generated PA images for the CASIA dataset (**a**) using hand-crafted-based face-PAD system [50] and (**b**) using deep-learning-based face-PAD system [15].

Supposing that the two distributions are Gaussian-like, the distance between the two distributions (padD) was measured as 5.463 for the deep-learning-based face-PAD system and 2.474 for the handcrafted-based face-PAD system. This result indicates that the deep-learning-based face-PAD method works well in detecting PA samples in the CASIA dataset. Because we are measuring the padD value for two different types of images, i.e., real and PA images, the measured padD indicates the distance between two different image domains. We see that the padD values in this experiment are much larger than those obtained using the captured and generated PA images in the above experiments (0.610 for the handcrafted-based and 0.711 for the deep-learning-based face-PAD system). This result indicates that the generated PA images have similar characteristics to the captured PA images in the CASIA dataset. We summarize our experimental results in Table 8. As the final experiment in this section, we measured the attack presentation classification error rate (APCER) of the generated PA images using the face-PAD system. By definition, the APCER indicates the proportion of PA images that were incorrectly classified as real images by a face-PAD system. In other words, the APCER represents the possibility of an attack successfully circumventing a face-PAD system. As a result, by measuring the APCER value, we can estimate the quality of generated PA images. The experimental results are shown in Figure 9 and Table 9. 

As shown in Figure 9, the distributions of captured real and generated PA images are quite far from each other and similar to those in Figure 8. In detail, the padD value for the deep-learning-based face-PAD system is approximately 6.745 and that for the handcrafted-based face-PAD system is approximately 3.128. These values are similar to those using the captured PA images (5.463 and 2.474, respectively). As shown in Table 9, we obtained APCERs of 9.488% and 4.292% for the captured PA and generated PA images, respectively, using the handcrafted-based face-PAD system. 

Using the deep-learning-based face-PAD system, we obtained APCER values of 0.784% and 0.000% using the captured PA and generated PA images, respectively. The APCER values produced by the handcrafted-based face-PAD system are much larger than those produced by the deep-learning-based system, which is caused by the fact that the deep-learning-based feature extraction method works much better than the handcrafted-based feature extraction method. By comparing the experimental results for the captured and generated PA images, we see that our approach generates PA images that contain the characteristics of PA images.

#### 4.2.3. Quality Assessment of Generated Images Using padD Measurement on Replay-Mobile Dataset

**Figure 10:**
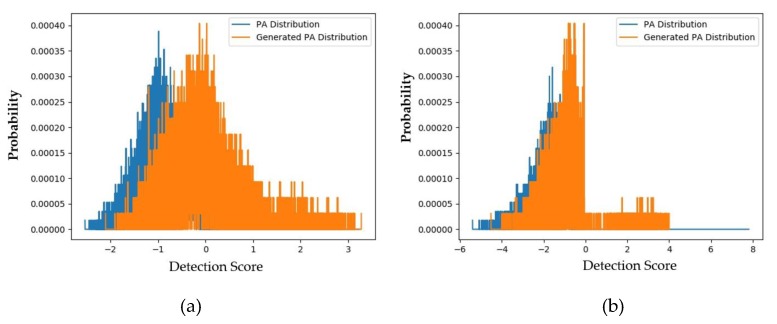
Distribution of detection scores of captured PA images versus the generated PA images with the Replay-mobile dataset (**a**) using the handcrafted-based face-PAD method [50]; (**b**) using the deep-learning-based face-PAD method [15].

**Figure 11:**
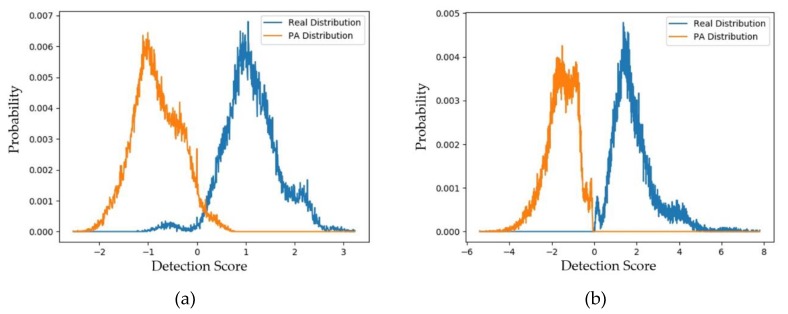
Distribution of detection scores of captured real versus captured PA images (**a**) using the handcrafted-based face-PAD method [50] and (**b**) using the deep-learning-based face-PAD method [15].

Similar to the experiments on the CASIA dataset, we performed experiments for the Replay-mobile dataset using the face-PAD systems. First, we measured the distributions and padD values for the use of captured PA versus generated PA images and the use of captured real and PA images. The experimental results of these experiments are given in Figure 10 and Figure 11 and Table 10. Figure 10 shows the distributions of the captured PA and generated PA images of the testing dataset. Similar to the experiments on the CASIA dataset described above, the two distributions (captured and generated PA images) are close to each other. In detail, the padD value for the deep-learning-based face-PAD system is approximately 0.836, and that for the handcrafted-based face-PAD system is approximately 1.214. 

Figure 11 shows the distribution of the scores of the captured real and captured PA images. For the deep-learning-based face-PAD system, we obtained a padD value of 3.928, and for the handcrafted-based face-PAD system, we obtained a padD value of 3.649. It is clear that these padD values are much larger than those produced by the captured and generated PA images. Through these results, we can conclude that the generated PA images are close to the captured PA images, while they are far from the captured real face images. In addition, we can see that the distributions of these two types of images do not overlap. This means that although the generated images have similar characteristics to the captured PA images, they are not identical, and the generated images can complement the captured PA images to fill the gap of missing PA samples.

**Figure 12:**
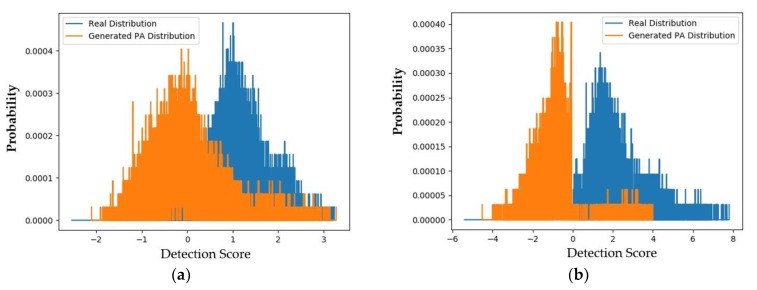
Distribution of detection scores of captured real and generated PA images for the Replay-mobile dataset (**a**) using the handcrafted-based face-PAD method [50] and (**b**) using the deep-learning-based face-PAD method [15].

In a subsequent experiment, we measured the APCER of the face-PAD systems using generated PA images. Figure 12 shows the distribution of detection scores of captured real and generated PA images for the deep-learning-based and handcrafted-based face-PAD systems. Similar to Figure 11, the distributions of the real and generated images are relatively separate. 

In detail, the two distributions obtained using the handcrafted-based face-PAD system have a padD value of 1.949, and those obtained using the deep-learning-based face-PAD system have a padD value of 3.211. This high padD value indicates that the generated PA images are different from the captured real face images. 

Table 11 lists the APCERs obtained in this experiment. Originally, the APCERs were 5.684% and 0.000% for the handcrafted-based and deep-learning-based face-PAD systems, respectively, using the captured data. Using the generated data, these APCER values increased to 41.294% and 1.551%. Although the error caused by the generated PA images in the handcrafted-based face-PAD system is much increased, the error caused by the generated PA images in the deep-learning-based face-PAD system is small. This is caused by the fact that the deep-learning-based method uses a deep CNN-RNN method for feature extraction, which results in higher performance than the handcrafted method. As shown in Figure 12b, the generated PA images have different characteristics to the real images. From this result and the results obtained using the CASIA dataset, we can conclude that the generated images efficiently captured PA features.

We presented our results using the CASIA dataset. Similarly, we presented our results using Replay-mobile dataset. As indicated in these experimental results, the APCER scores of generated PA images are lower than captured PA images, but APCER scores of generated PA images are higher than captured PA ones. The reason for this result is that we trained our PA image generation model using two different datasets which have slightly different characteristics and the amount of PA images. As explained at the beginning of Section 4, the CASIA dataset contains real and PA images of 50 people using various attack methods, including three levels of image quality (low, normal, and high), and three methods for making PA samples (using cut-photo, wrap-photo, and video). Compared to the CASIA dataset, the Replay-mobile dataset only contains PA images for the photo and video attack using a mobile camera. As indicated in the previous study [15], the CASIA dataset has higher complexity of PA images than Replay-mobile dataset, which is indicated by the fact that ACER of an up-to-date face-PAD system [15] is approximately 1.286% and 0.0015% for the CASIA and Replay-mobile dataset, respectively. Because of this reason, we obtained a face-PAD system which covers various kinds of PA images using CASIA dataset (the effects of a new type of PA images on the face-PAD system is small). However, the face-PAD system is more affected by noise and new kind of PA images when it is trained by Replay-mobile dataset because this dataset has limited types of PA images (the effects of a new type of PA images is large). As a result, the APCER of generated PA images is small in the experiment with CASIA dataset, and high in the experiment with Replay-mobile dataset.

#### 4.2.4. Processing Time of the Proposed Approach

**Figure 13:**
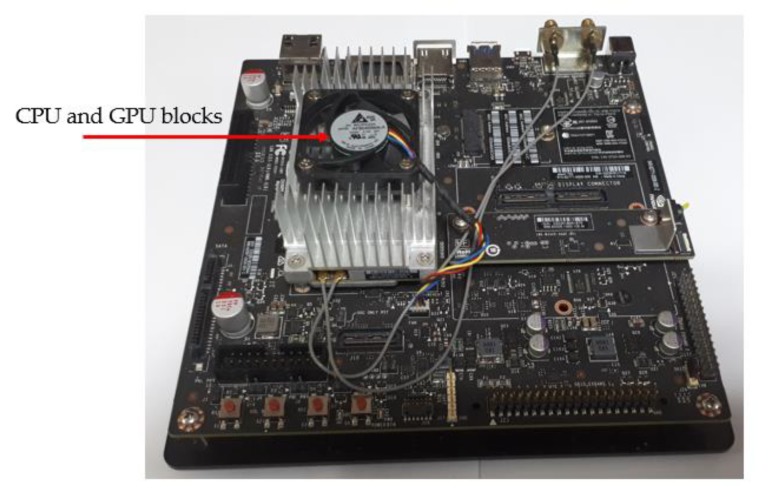
NVIDIA Jetson TX2 on-board embedded system.

As a final experiment, we measured the processing time of our proposed method for generating PA images using the pretrained model to investigate the running speed of our approach. In our experiments, we ran our generation model in two different hardware systems: a general-purpose computer and an embedded system based on the NVIDIA Jetson TX2 board [52]. First, we used a general-purpose computer with an Intel Core i7 central processing unit (CPU) (Intel Corporation, Santa Clara, CA, USA) and 64 GB of RAM. For the deep-learning-based image generation model, we used a TitanX graphics processing unit (GPU) card [53] and the Tensorflow library [54] as the running environment. As the second option, we ran our image generation model on an NVIDIA Jetson TX2 embedded board, as shown in Figure 13. This is a popular deep-learning-based embedded system developed by NVIDA Corporation, which integrates both the CPU and GPU for deep-learning purposes and has been used for on-board deep-learning processing in self-driving cars. For running a deep-learning-based model, the Jetson TX2 board has an NVIDIA PascalTM-family GPU (256 CUDA cores) with 8 GB of memory shared between the CPU and GPU and 59.7 GB/s of memory bandwidth. Because this board is designed for an embedded system, it uses less than 7.5 W of power. The experimental results are given in Table 12. As shown in this table, it took approximately 29.920 ms to generate a PA image using the general-purpose computer. This means that our generation model can run at a speed of 33.4 frames per second (fps). Using the Jetson TX2 embedded system board, it took approximately 62.423 ms to generate a PA image, which corresponds to 16.02 fps. Compared to the processing time offered by the desktop computer, the Jetson TX2 embedded systems required longer processing time due to its limited computation resources compared to a general-purpose computer. However, with a speed of 16.02 fps with the embedded system and 33.4 fps with the general-purpose computer, we can conclude that our approach is relatively fast and sufficient to run both in general and in embedded environments.

## 5. Conclusions

In this paper, we proposed a method for generating PA face images for face-PAD systems. We trained a generation model based on the CycleGAN method using images from two domains, i.e., captured real face images and captured PA images, to learn the characteristics of images in each class and the relations between these two classes. As a result, we showed that the generated PA images are quite similar to but do not overlap with captured PA images, which were collected using a conventional camera and attacking methods. Because the generated images are not identical to the captured PA images, we hope that they can fill the gap of missing samples caused by the lack of PA images because of the diversity of attack methods. 

In this study, we aimed to generate PA images to reduce the efforts required for simulating presentation attack methods and PA image acquisition procedure. Therefore, we used a fusion of all kinds of PA images in our experiments without considering every single attack method. Even we can train image generation model using PA images of single available presentation attack method (print attack, display attack etc.), this scheme has some limitations that make it not suitable for our research purpose. First, training an image generation model for every single attack method results in multiple generation models for a single problem. As a result, it wastes processing time, storage, and makes the system complex. Second, as we have explained, the presentation attack detection problem has a special property that we cannot simulate all possible attack methods because of various types of presentation attack instruments and attacking procedures. Therefore, the use of a fusion of existing PA images helps to learn the characteristics of PA images in general to simulate an unknown attack method. In our future work, we plan to use generated images along with captured images to train a face-PAD system to validate the efficiency of the generated images and also to reduce the error of the face-PAD system.

## Figures and Tables

**Table 1:** Summary of GAN-based image generation methods for biometrics systems.

Task	Purpose
Fingerprint image generation [28,33]	-Generate realistic fingerprint images that are close to captured fingerprint images [28,33]. -Demonstrate that synthetic fingerprint images are capable of spoofing multiple peoples’ fingerprint patterns [33].
Iris image generation [27]	-Generate realistic iris images that are close to captured iris images
Palm-print image generation [26]	-Generate realistic palm-print images and use them as augmented data to train a palm-print recognition system. -Enhance the performance of a palm-print recognition system using generated images.
PA face image generation (Our approach)	-Generate realistic PA face images to reduce the effort required for image acquisition. -Fill the gap of missing samples caused by diversity of attack methods.

**Table 2:** Detailed description of the discriminator network used in our study (input and output shape is in format: height × width × depth).

Type of Layer	Kernel Size	Stride	Number of Filters	Alpha of Leaky ReLU	Input Shape	Output Shape
Convolution	4 × 4	2	64	-	256 × 256 × 3	128 × 128 × 64
Leaky ReLU	-	-	-	0.2	128 × 128 × 64	128 × 128 × 64
Convolution	4 × 4	2	128	-	128 × 128 × 64	64 × 64× 128
Instance Normalization	-	-	-	-	64 × 64 × 128	64 × 64 × 128
Leaky ReLU	-	-	-	0.2	64 × 64 × 128	64 × 64 × 128
Convolution	4 × 4	2	256	-	64 × 64 × 128	32 × 32 × 256
Instance Normalization	-	-	-	-	32 × 32 × 256	32 × 32 × 256
Leaky ReLU	-	-	-	0.2	32 × 32 × 256	32 × 32 × 256
Convolution	4 × 4	1	512	-	32 × 32 × 256	32 × 32 × 512
Instance Normalization	-	-	-	-	32 × 32 × 512	32 × 32 × 512
Leaky ReLU	-	-	-	0.2	32 × 32 × 512	32 × 32 × 512
Convolution	4 × 4	1	1	-	32 × 32 × 512	32 × 32 × 1

**Table 3:** Detailed description of the generator network used in our study (input and output shape is in format: height × width × depth).

Type of Layer		Block	Kernel Size	Stride	Number of Filters	Input Shape	Output Shape
**Convolution**		Encoding	7 × 7	1	64	256 × 256 × 3	256 × 256 × 64
Instance Normalization			-	-	-	256 × 256 × 64	256 × 256 × 64
ReLU			-	-	-	256 × 256 × 64	256 × 256 × 64
Convolution			3 × 3	2	128	256 × 256 × 64	128 × 128 × 128
Instance Normalization			-	-	-	128 × 128 × 128	128 × 128 × 128
ReLU			-	-	-	128 × 128 × 128	128 × 128 × 128
Convolution			3 × 3	2	256	128 × 128 × 128	64 × 64 × 256
Instance Normalization			-	-	-	64 × 64 × 256	64 × 64 × 256
ReLU			-	-	-	64 × 64 × 256	64 × 64 × 256
Residual (Repeated 9 times)	Convolution		3 × 3	1	256	64 × 64 × 256	64 × 64 × 256
	Convolution		3 × 3	1	256	64 × 64 × 256	64 × 64 × 256
Deconvolution		Decoding	3 × 3	2	128	64 × 64 × 256	128 × 128 × 128
Instance Normalization			-	-	-	128 × 128 × 128	128 × 128 × 128
ReLU			-	-	-	128 × 128 × 128	128 × 128 × 128
Deconvolution			3 × 3	2	64	128 × 128 × 128	256 × 256 × 64
Instance Normalization			-	-	-	256 × 256 × 64	256 × 256 × 64
ReLU			-	-	-	256 × 256 × 64	256 × 256 × 64
Convolution			7 × 7	1	3	256 × 256 × 64	256 × 256 × 3
Tanh Normalization			-	-	-	256 × 256 × 3	256 × 256 × 3

**Table 4:** Description of the original CASIA dataset used in our experiments.

CASIA Dataset	Training Dataset (20 Persons)		Testing Dataset (30 Persons)		Total
	Real Access	PA Access	Real Access	PA Access	
Number of Videos	60	180	90	270	600
Number of Images	10,940	34,148	16,029	49,694	110,811

**Table 5:** Description of the original Replay-mobile dataset used in our experiments.

Replay-Mobile Dataset	Training Dataset (12 Persons)		Testing Dataset (12 Persons)		Total
	Real Access	PA Access	Real Access	PA Access	
Number of Videos	120	192	110	192	614
Number of Images	35,087	56,875	32,169	56,612	180,743

**Table 6:** FID measurements for the captured images versus generated images using our proposed method in comparison with intra-class FID and the model based on conventional cross-entropy loss function.

FID Measurement	Using CASIA Dataset	Using Replay-Mobile Dataset
Intra-class FID	24.614	37.943
Inter-class FID using CycleGAN-based model with conventional cross-entropy loss function	30.968	51.207
Inter-class FID by our proposed method (using least-squared loss function)	28.300	42.066

**Table 7:** Comparison of the measured FIDs in our study with those achieved in previous studies.

Method	DCGAN for Generation				CycleGAN for Generation	
	Iris Images [27]	Fingerprint Images [28]	Replay-Mobile Dataset	CASIA Dataset	Replay-Mobile Dataset	CASIA Dataset
FID	41.08	70.5	65.049	82.400	42.066	28.300

**Table 8:** PadD measurements of generated PA images using the CASIA dataset.

Handcrafted-Based PAD Method [50]		Deep-Learning-Based PAD Method [15]	
Captured PA versus Generated PA Images	Captured Real versus Captured PA Images	Captured PA versus Generated PA Images	Captured Real versus Captured PA Images
0.610	2.474	0.711	5.463

**Table 9:** APCERs of PA images using different face-PAD methods on CASIA dataset (unit: %).

Handcrafted-Based Face-PAD Method [50]		Deep-Learning-Based Face-PAD Method [15]	
Captured PA Images	Generated PA Images	Captured PA Images	Generated PA Images
9.488	4.292	0.784	0.000

**Table 10:** padD measurements of generated PA images using Replay-mobile dataset.

Handcrafted-Based Face-PAD Method [50]		Deep-Learning-Based Face-PAD Method [15]	
Captured PA versus Generated PA Images	Captured Real versus Captured PA Images	Captured PA versus Generated PA Images	Captured Real versus Captured PA Images
1.214	3.649	0.836	3.928

**Table 11:** APCERs of PA images using different face-PAD methods (unit: %).

Handcrafted-Based Face-PAD Method [50]		Deep-Learning-Based Face-PAD Method [15]	
Captured PA Images	Generated PA Images	Captured PA Images	Generated PA Images
5.684	41.294	0.00	1.551

**Table 12:** Processing time of proposed method on a desktop general purpose computer and a Jetson TX2 embedded system (unit: ms).

Desktop Computer	Jetson TX2 Embedded System
29.920	62.423
